# Thyroid hormone-mediated autophagy and mitochondrial turnover in NAFLD

**DOI:** 10.1186/s13578-016-0113-7

**Published:** 2016-07-19

**Authors:** Rohit Anthony Sinha, Paul M. Yen

**Affiliations:** Laboratory of Hormonal Regulation, Cardiovascular and Metabolic Disorders Program, Duke-NUS Graduate Medical School, 8 College Road, Singapore, 169857 Singapore

**Keywords:** Autophagy, Liver, Mitophagy, NAFLD, Thyroid hormones, ULK1

## Abstract

Non-alcoholic fatty liver disease (NAFLD) is a fast-growing silent epidemic that is present in both developed and developing countries. Initially thought as a benign deposition of lipids in the liver, it now has been shown to be a major risk factor for type II diabetes and one of the leading causes of cirrhosis. Recent findings suggest that dysregulation of mitochondrial homeostasis and autophagy play critical roles in the hepatocyte injury and insulin resistance of NAFLD. Thyroid hormone (TH) is a major stimulator of hepatic autophagy and mitochondrial function. Decreased TH action has been associated with NAFLD in man. In this review, we highlight some of the new discoveries that demonstrate the roles of TH in hepatic mitochondrial homeostasis via mitophagy and their implications for NAFLD.

## Background

Non-alcoholic fatty liver disease (NAFLD) is initiated by lipid accumulation in hepatocytes [1, 2] that leads to a spectrum of liver dysfunction ranging from excess lipid storage in the liver (hepatosteatosis) to progressive non-alcoholic steatohepatitis (NASH), that in turn, increases the risk for cirrhosis and hepatocellular cancer. NAFLD occurs in 25–35 % of the general US. population, and its prevalence is estimated to be 60–80 % in patients with type II diabetes milletus (DM) and obesity [2]. In addition to causing hepasteatosis and inflammation within the liver, NAFLD also can have profound metabolic effects by inducing hepatic insulin resistance [3]. Additionally, defects in β-oxidation of fatty acids and lipotoxicity owing to intracellular over-accumulation of fatty acids and their toxic metabolites are thought to play important roles in the pathogenesis of NAFLD [1, 2]. The progression in NAFLD often leads to insulin resistance, increased hepatic glucose production, and worsened glycemic control in diabetic patients, resulting in a vicious cycle that further exacerbates the manifestations and complications of diabetes. Unfortunately, little is known about hormonal regulation of hepatosteatosis and gluconeogenesis in NAFLD or the roles of hormones in disease progression. Moreover, although there are many drug therapy options for treating hyperglycemia in diabetes; currently, there are no effective drug treatments for NAFLD.

Thyroid hormones (THs: T_3_, T_4_) promote the oxidation of fatty acids within the liver, so it is possible that impaired TH action in the liver may contribute to NAFLD. Indeed, it recently has been shown that the incidence of NAFLD is doubled in patients with hypothyroidism [4] with approximately 15 % patients affected. Additionally, T_3_ and several TH analogs can ameliorate NAFLD in rodents that are fed high fat diet (HFD) [5, 6]. At the genomic level, many of the genes that have altered expression in NAFLD are regulated by TH [7], further supporting the notion that defects in TH signaling may promote hepatosteatosis and hepatic damage. Two recent studies also have shown that thyroid hypofunction occurs with higher frequency in both young and elderly adults with NAFLD [8, 9].

T_3_ stimulates the conversion of triglycerides to free fatty acids for delivery into the mitochondria by increasing mRNA expression and activities of hepatic lipases [10]. While this process is well described, it is possible that other cellular pathways may be involved in the delivery of stored triglycerides from lipid droplets to mitochondria. Recently, autophagy has been shown to promote cell survival during nutrient deprivation and upon exposure to inflammatory or pro-apoptotic stimuli [11, 12]. Moreover, autophagy has been implicated in the direct catabolism of fatty acids through “lipophagy” [13] and inhibition of autophagy leads to the development of fatty liver and insulin resistance [14]. We previously showed that T_3_ stimulated hepatic fatty acid oxidation through lipophagy [15]. We and others also have showed that T_3_ and TH analogs can decrease hepatosteatosis in cell culture and in rodent models [5, 6, 15]. The accompanying increase in oxidative phosphorylation leads to increased mitochondrial reactive oxygen species (ROS) production that can cause mitochondrial damage and cell death [16].

## TH stimulates mitophagy and mitochondrial biogenesis

**Fig. 1:**
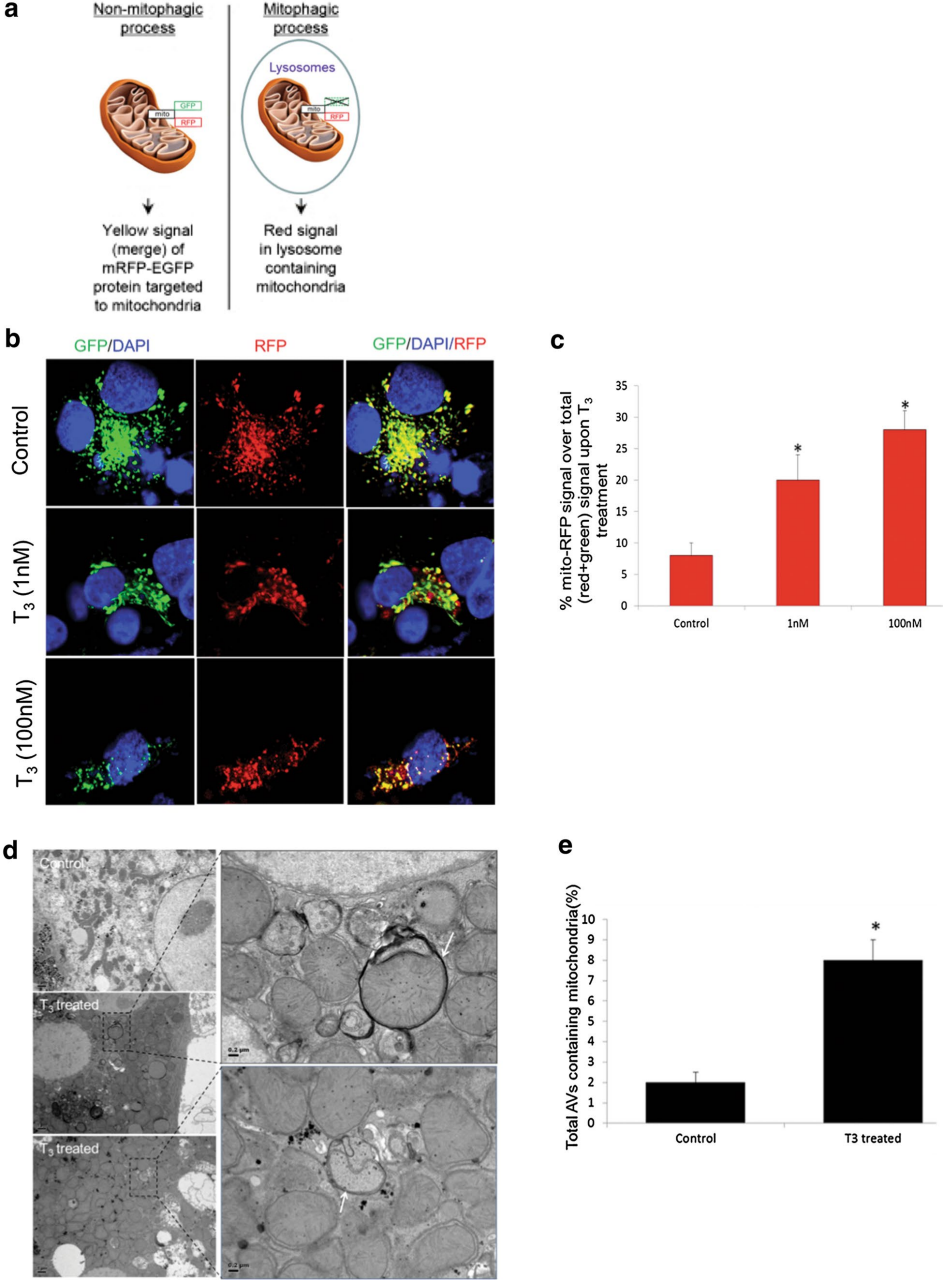
T_3_ increases autophagy and mitophagy in hepatic cells. **a** Model showing how mitochondria-specific mRFP-GFP protein detects mitophagy. **b** Monitoring mitophagic flux using dual fluorescence p-mito-mRFP-EGFP reporter (pAT016) in HepG2 cells. Lysosomal delivery of the tandem fusion protein Mito-mRFP-EGFP along with entire mitochondria results in differential quenching and degradation of the two individual fluorochromes, thus allowing for visual analysis of mitophagic flux. TRβ1-HepG2 cells transiently expressing Mito-mRFP-EGFP were treated with 1 nM or 100 nM T_3_ for 48 h followed by visualization using confocal microscopy (40× magnification). Nuclei were stained with DAPI (*blue*). In the images, fluorescence signals indicates the expression of Mito-mRFP-EGFP targeting mitochondria: *yellow color* no mitophagy or normal cytosolic mitochondria, *red color* mitophagy or mitochondria inside lysosomes. **c** Quantitative analysis of the RFP (red-only) fluorescence to denote % mitophagy was done. Quantification of images (at least 20 transfected cells per each sample in 3 different fields) was conducted with ImageJ software. *Bars* represent the mean of the respective individual ratios ± SD (*p < 0.05). **d** Electron micrograph of primary mouse hepatocytes treated with T_3_. EM of untreated control and T_3_-treated (100 nM/24 h) mouse hepatocytes showing increased mitophagy (Denoted by *arrows* showing autophagosomes containing mitochondria) under T_3_ treatment. *Scale bar* 1 µm and in enlarged figures are 0.2 µm. **e** *Bar graphs* showing % of autophagosomes (AVs) containing mitochondria in control and T_3_-treated primary mouse hepatocytes based on EM micrograph images. Scoring was done by counting 10–15 different autophagic vesicles in 5 random fields per condition (n = 3, *p < 0.05. Adapted from Ref. [16], Figs. 4 and 6

The major mechanisms for mitochondrial repair are mitochondrial fusion, fission, and mitochondrial autophagy or “mitophagy” [17]. To determine the occurrence of mitophagy, we used a tandem-tagged RFP-EGFP chimeric plasmid, pAT016, encoding a mitochondria targeting signal sequence fused in-frame with RFP and EGFP genes (tandem tagged Mito-mRFP-EGFP). RFP and GFP have different stabilities in an acidic environment [18]. The GFP signal is quenched at lower pH whereas RFP can be visualized in acidic autolysosomes; thus, increased RFP/red-only fluorescence in the lysosomes indicates completion of the mitophagic process (Fig. 1a). Using this assay, we observed that T_3_ increased autolysosome-resident mitochondria (red fluorescent dots without any green fluorescence) at concentrations as low as 1 nM and as early as 24 h (Fig. 1b, c). We then conducted studies of autophagy in primary mouse hepatocytes. T_3_ induced autophagic flux and mitophagy in primary mouse hepatocytes observed mitochondrial protein accumulation and the presence of autophagosome-resident mitochondria by electron microscopy (Fig. 1d, e). These findings demonstrate that these cell-autonomous effects occurred in primary hepatic cells with normal TRβ expression. Similar effects for autophagic flux also were seen in cell culture and in mice treated with the lysosomal inhibitor, chloriquine.

**Fig. 2:**
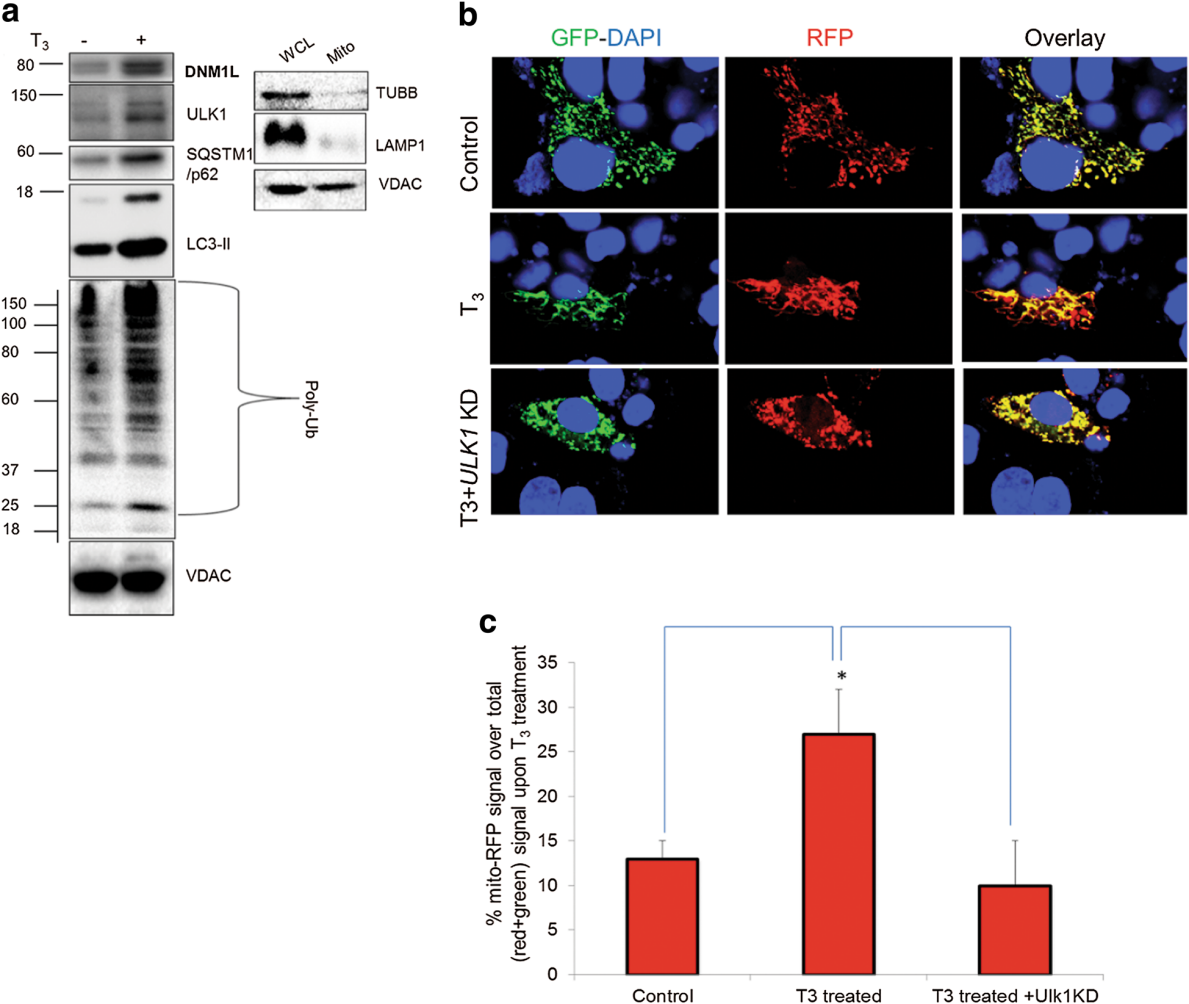
Mitophagy proteins translocate to mitochondria and are necessary for T_3_ stimulation of mitophagy. **a** Immunoblot showing mitochondrial protein ubiquitination and localization of ULK1, p62, LC3-II, and Drp1 proteins in isolated mitochondrial fraction from T_3_ (100 nM/48 h)-treated TRβ1-HepG2 cells. Purity/enrichment of the mitochondrial fraction (Mito) was verified by the absence of β-Tubulin (cytosolic) and LAMP-1 (lysosomal) relative to its level in the whole cell lysate (WCL) for the same amount of VDAC levels. **b** TRβ-HepG2 cells transiently expressing Mito-mRFP-EGFP were treated with 100 nM T3 for 48 h with or without ULK1 KD followed by visualization using confocal microscopy (40× magnification). Nuclei were stained with DAPI (*blue*). In the images, fluorescence signals indicate the expression of Mito-mRFP-EGFP targeting mitochondria: *yellow color* no mitophagy, *red color* mitophagy. **c** Quantitative analysis of the RFP (*red*) fluorescence to denote  % mitophagy. Quantification of images (at least 10 transfected cells per each sample in 3 different fields) was conducted with ImageJ software. *Bars* represent the mean of the respective individual ratios ± SD (*p < 0.05). Adapted from Ref. [16], Fig. 9

Mitochondrial translocation of the autophagic machinery is required for mitophagy so we measured the levels of autophagic proteins in purified mitochondrial fractions that were verified to be free from cytosolic and lysosomal contamination (Fig. 2a). T_3_ treatment increased the localization of Ubiquitin-like protein 1 (ULK1), p62, and LC3II within the mitochondrial fraction of HepG2 cells. Dynamin 1-like protein (Drp1), a protein associated with mitochondrial fission and mitophagy also was preferentially recruited to mitochondria after T_3_ treatment (Fig. 2a). Additionally, increased mitochondrial protein ubiquitination was observed in T_3_-treated cells (Fig. 2a) consistent with the notion that mitochondrial ubiquitination precedes mitophagy. Confocal imaging of mt-RFP-EGFP in conjunction with the mitochondrial marker, TOMM20, showed that T_3_ increased mitophagy. However, treatment with ULK1 siRNA decreased mitophagy induced by T_3_ back to baseline level. Thus, mitophagy critically depends upon ULK1 and suggests that the latter is a necessary component for forming the nascent autophagosome that engulfs mitochondria (Fig. 2b, c) [16]. Interestingly, siRNA knockdown of ULK1 did not abrogate general autophagy suggesting that, unlike mitophagy, this process might be complemented by another isoform of ULK, ULK2 [16].

We observed induction of hepatic mitochondria biogenesis by T_3_-mediated stimulation of PGC1a and mitochondrial protein expression. The latter proteins increased their accumulation when autophagy was blocked suggesting that there was increased mitochondrial turnover involving both mitophagy and mitochondrial synthesis. The transcriptional expression of several genes involved in mitophagy, Bnip, Nix, ULK1, p62, and LC3 mRNAs also were induced by T_3_. Additionally, the master regulator of autophagy and lysosomal genes, Transcription factor EB (TFEB) as well as PGC1a, Tfam, and Cox 4 mRNAs were induced by T_3_.

## Tissue-specific hypothyroidism in NAFLD

We examined livers from mice fed a methionine and choline deficient (MCD) diet for 12 weeks and found that the highly sensitive TH-responsive gene, Deiodinase 1 (DIO1), a deiodinase enzyme that converts T_4_ to T_3_, was significantly reduced in livers of MCD-fed rats compared to livers from control animals fed normal chow diet. Moreover, the MCD fed group exhibited grade 2 steatohepatitis on histology. We then measured intrahepatic T_3_, T_4_, and rT_3_ concentrations in livers from MCD-fed rats vs. rats fed normal chow diet. For the MCD fed rats, hepatic T_3_ concentration was significantly decreased, rT_3_ whereas hepatic T_4_, and rT_3_ concentrations were not changed (Sinha and Yen, unpublished data). In pilot studies in these rats, we found that DIO1 as well as OATP1 and MCT8 (thyroid hormone transporters) mRNA expression were decreased suggesting that intrahepatic hypothyroidism may be a feature of, as well as a contributor towards, the development of NASH in these rats.

Consistent with our data, two previous studies showed that T_3_ or TH analogs decreased hepatosteatosis in mouse and rat models [5, 6]. In order to assess whether T_3_ decreased lipotoxicity, a common feature of steatohepatitis, we examined the effects of TH on palmitate-induced cell death. Preliminary results showed palmitate markedly increased cleaved caspase 3 in TRβ-HepG2 cells, and this was attenuated by co-treatment with T_3_. Palmitate itself induced an increase in autophagy. However, the improvement in cell survival provided by T_3_ also was accompanied by a further increase in autophagy. Although palmitate increased oxidative phosphorylation as measured by the Seahorse XF Analyzer available in our laboratory, T_3_ further increased oxidative phosphorylation (Sinha and Yen, unpublished results.). These results suggest that induction of lipophagy and β-oxidation by T_3_ may protect against lipotoxicity due to toxic lipids derived from excessive intracellular palmitate such as such as ceramides or diacylglycerol. On the basis of the foregoing pre-clinical data, we recently have initiated a pilot clinical study on the effects of low dose levothyroxine therapy in reducing hepatic fat content and improving glucose control in diabetic Chinese male patients with hepatosteatosis. Patients are treated with low dose levothyroxine for 4 months, with their liver fat content measured both before and after treatment by MRI spectroscopy. Currently, we are in the middle of the enrollment of patients for our study.

## Role of SIRT1 on T_3_-mediated autophagy

We also have shown that a subset of forkhead box protein O1 (FOXO1) target genes, including those involved in gluconeogenesis, are co-regulated by T_3_ through its metabolic activation of SIRT1 and its regulation of downstream deacetylation and dephosphorylation of FOXO1 [19, 20]. SIRT1 is a deactylase that can be activated by increased NAD+ concentration, and thus can act as an intracellular energy sensor to modulate the transcriptional activity by both TH and FOXO1 [21]. Besides its critical role in gluconeogenesis, we also have found that SIRT1 is required for TH-mediated autophagy by virtue of its ability to stimulate the expression of target genes involved in autophagy as well as deacetylate ATG proteins. Thus, SIRT1 plays vital roles in in initiating and maintaining the autophagy that is required for β-oxidation of fatty acids as well as mitophagy by TH. In this connection, mitochondria degeneration has been associated with metabolic disorders and aging. Thus, the maintenance of normal mitophagy may be a crucial aspect in preventing cell death in tissues such as the pancreas and liver in diabetes. Furthermore, it is possible that maintaining tissue-specific euthyroidism may promote normal lipid metabolism as well as preserve mitochondrial function in the liver. Indeed, the expression of target genes involved in lipid metabolism that are regulated by TH are decreased in liver samples from patients undergoing bariatric surgery [7].

## Conclusions

In summary, we believe that our studies to elucidate the mechanisms of hepatic mitochondrial turnover by TH and SIRT1 and the role of autophagy in NAFLD, will lead to better understanding of the role of hormones and their potential dysregulation in the pathogenesis and progression of this condition. This information could lead to better diagnosis and treatments for NAFLD as well as other metabolic disorders.

## Abbreviations

Drp1: dynamin 1-like protein
FOXO1: forkhead box protein O1
MCD: methionine and choline deficient
NAFLD: non-alcoholic fatty liver disease
NASH: non-alcoholic steatohepatitis
TH: thyroid hormones
ULK1: ubiquitin-like protein 1
